# Crosstalk between gut microbiota and sepsis

**DOI:** 10.1093/burnst/tkab036

**Published:** 2021-10-26

**Authors:** Mengwei Niu, Peng Chen

**Affiliations:** Department of Pathophysiology, Guangdong Provincial Key Laboratory of Proteomics, School of Basic Medical Sciences, Southern Medical University, Guangzhou 510515, China; Department of Pathophysiology, Guangdong Provincial Key Laboratory of Proteomics, School of Basic Medical Sciences, Southern Medical University, Guangzhou 510515, China

**Keywords:** Sepsis, Microbiota, Immune, Gut barrier, Liver, Fecal microbiota transplantation, Probiotics

## Abstract

Sepsis is an overwhelming inflammatory response to microbial infection. Sepsis management remains a clinical challenge. The role of the gut microbiome in sepsis has gained some attention. Recent evidence has demonstrated that gut microbiota regulate host physiological homeostasis mediators, including the immune system, gut barrier function and disease susceptibility pathways. Therefore, maintenance or restoration of microbiota and metabolite composition might be a therapeutic or prophylactic target against critical illness. Fecal microbiota transplantation and supplementation of probiotics are microbiota-based treatment methods that are somewhat limited in terms of evidence-based efficacy. This review focuses on the importance of the crosstalk between the gastrointestinal ecosystem and sepsis to highlight novel microbiota-targeted therapies to improve the outcomes of sepsis treatment.

Highlights

- Sepsis potentially disrupts the gut ecosystem and gut dysbiosis may predispose sepsis development.
- Gut barrier function and the gut–liver axis play essential roles in the host response to sepsis.
- In addition to supplementation with probiotics and prebiotics, which have been shown to be clinically effective, fecal microbiota transplantation and phage therapy are promising therapeutic options for the future.

## Background

Sepsis is the manifestation of a dysregulated host response, characterized by organ dysfunction, to overwhelming infection. Even with optimal management in intensive care units, sepsis is associated with high mortality rates and the World Health Organization has recognized sepsis as a global health emergency [1]. Excessive inflammation can eventually lead to dysfunction of multiple organ systems, including the pulmonary, hepatic, cardiovascular, renal and gastrointestinal systems [2]. Epidemiological studies have revealed high rates of sepsis and in-hospital mortality ranging from 25 to 30% [3]. In clinical practice, the Sequential Organ Failure Assessment (SOFA) score is used to evaluate organ dysfunction [4]. Given that sepsis is life-threatening, effective strategies to prevent, diagnose and treat it are essential.

Sepsis occurs due to impaired activation of the immune system in response to infection; therefore, its onset depends on microbial pathogen-associated molecular patterns (PAMPs) that are recognized by pattern-recognition receptors (PRRs). Meanwhile, damage-associated molecular patterns (DAMPs) derived from body tissues, such as mitochondrial DNA, heat shock proteins and high mobility group box 1 protein (HMGB1), act as potent activators of the innate immune system and initiate a systemic reaction [5].

The host immune reaction during and after sepsis onset is complicated. Hyperactivation of immune cells and overwhelming inflammation appear to be the main contributors to the pathophysiology of sepsis [6]. These processes induce the release of both proinflammatory factors and anti-inflammatory mediators through the activation of inflammatory signaling pathways. The overproduction and release of cytotoxic molecules, such as interleukin (IL)-1, IL-17 and tumor necrosis factor (TNF), which constitute the cytokine storm, are likely responsible for the excessive systemic inflammation associated with sepsis [6]. However, sepsis is also associated with immune suppression, which is mediated by both the adaptive and innate immune systems [7]. Immune suppression often increases the individual’s susceptibility to secondary infections, further increasing the risk of death [8]. Furthermore, there are several other key mechanisms, such as the complement pathway, Ca^2+^ homeostasis and mitochondrial dysfunction, that affect immune function during sepsis [6]. The precise series of mechanisms underlying sepsis-induced multiple organ dysfunction are not fully understood, and new insights into these processes will facilitate the development of novel therapeutics to improve sepsis outcomes.

**Figure 1.**
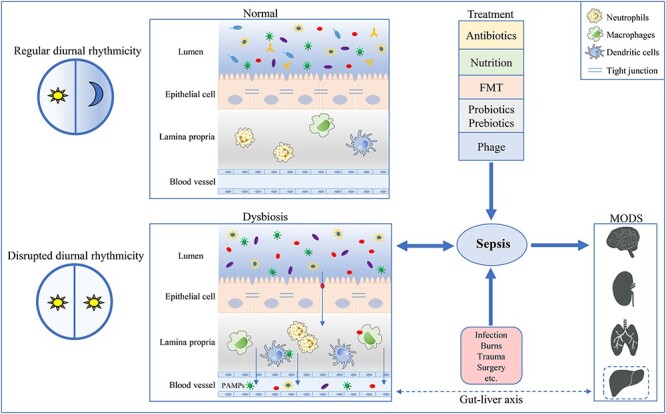
The relationship between gut microbiota and sepsis. Intestinal epithelial cells form a physical and chemical barrier to prevent bacteria and bacterial products from translocating into the lamina propria and systemic circulation. Sepsis results in gut barrier dysfunction and microbiome composition alteration, and gut bacterial dysbiosis predisposes sepsis development. Additionally, the gut–liver axis plays an important role in sepsis. Circadian disruption could cause gut microbial alterations, which possibly impact sepsis development. Promising approaches to sepsis treatment include FMT and supplementation with probiotics or prebiotics. *FMT* fecal microbiota transplantation, *PAMPs* pathogen-associated molecular patterns, *MODS* multiple organ dysfunction syndrome

The human gastrointestinal tract contains trillions of bacteria, the composition of which mediates the equilibrium between host wellness and disease. The gut epithelial barrier, immune system and gut microbiome are all closely interlinked to defend the body against pathogen colonization [9]. The gut microbiome has been well studied via metagenome sequencing and 16S ribosomal DNA sequencing. Gastrointestinal microbiome diversity and function have been implicated in several disorders, such as *Clostridioides difficile* infection (CDI), inflammatory bowel disease (IBD) and liver disease [10,11]. An imbalance of the microbiome profile has been termed dysbiosis. The gut has been recognized as the primary target site of many diseases, as gut homeostasis is among the most commonly affected physiological elements. The gut microbiome is reported to drive severe responses to sepsis and affect the outcomes of sepsis treatment [12]. Multi-omics analysis has revealed that compositional and functional alteration of the gut microbiota promotes sepsis-linked organ damage [13]. A prospective cohort study indicated that gut dysbiosis with accumulation of bacilli and their fermentation metabolites could precede late-onset sepsis [14]. The detailed pathogenetic role of gut microbiota in sepsis is incompletely understood. Here, we review the character of the gut microbiome in terms of its association with sepsis and provide an overview of potential treatments for sepsis (Figure 1).

## Review

### Sepsis may aggravate gut microbiome dysbiosis

It has been reported that critically ill patients who suffer sudden and severe insults present with immediate and dramatic gut microbiota alterations, along with decreased concentrations of short-chain fatty acids (SCFAs) in the gut [15]. A prospective observational study adopted shotgun metagenomic sequencing, phylogenetic profiling and microbial genome analyses to confirm that antibiotics administration in the intensive care unit markedly decreases gut microbiota diversity and that the elimination of commensal strains facilitates the transmission of hospital-acquired *Enterococcus faecium* infection [16]. Previous research has revealed that severe systemic inflammatory response syndrome is associated with a markedly decreased abundance of beneficial *Bifidobacterium* and *Lactobacillus* and increased levels of pathogenic *Staphylococcus* and *Pseudomonas* [17]. Clinically, the gut ecosystem may be a prognostic marker for patients with septic complications. Death of patients with systemic inflammatory response syndrome has been associated with a lower abundance of obligate anaerobes and a higher abundance of pathogenic bacteria relative to normal or baseline levels [18]. Commensal bacteria, particularly obligate anaerobes, play an important role in preventing pathogen invasion and colonization. These beneficial microbes inhibit the adherence of pathogenic bacteria to the intestinal epithelial brush border [19]. Conversely, overgrowth of pathogenic bacteria facilitates the progression of infection [17].

 Patients with sepsis have been shown to have lower levels of fecal SCFAs than healthy volunteers, and this reduced concentration has been shown to last for 6 weeks [20]. SCFA absorption is lower in association with critical illness, and this could aggravate gut barrier dysfunction, facilitate pathogen colonization and affect the systemic immune response [21]. SCFAs also provide energy for intestinal epithelial cells and influence the proliferation and differentiation of these cells [21]. Low SCFA concentrations may negatively impact host recovery from critical illness. By employing multi-omics analysis, we previously found septic patients to exhibit enteric dysbiosis at both the compositional and functional levels [13]. Importantly, such disrupted ecosystems could promote septic organ damage progression, further demonstrating that sepsis-associated dysbiosis may serve as an upstream contributor to sepsis development.

### Gut microbiota modulate sepsis progression

#### Gut microbiota influence the immune response during sepsis

The intestinal microbiome has been recognized as an important modulator of the host immune system. Commensal microorganisms act as essential factors in response to bacterial infection in distal tissues [22]; for example, translocation of peptidoglycan from the gut can directly enhance neutrophil function and rapidly primes the systemic immunomodulatory response to pneumococcal sepsis [23]. The clearance of gut bacteria by antibiotic treatment or the use of germ-free animals has been reported to influence inflammation and organ injury in sepsis [24,25]. Mechanistically, gut microbiota clearance impairs immune cell function. Macrophages obtained from gut microbe-depleted mice have been shown to express diminished phagocytic ability with increased bacterial dissemination and organ failure. Importantly, transcriptome analysis of alveolar macrophages has shown that the gut microbiome influences metabolic pathways, especially those responsible for responsiveness toward bacterial virulence factors and phagocytosis capacity [26]. The microbiome regulates the number of aged neutrophils, and a study of mice treated with antibiotics and injected with lipopolysaccharide (LPS) showed that the antibiotics significantly reduced the abundance of neutrophil extracellular traps [25]. Clinically, antibiotic use can alleviate pathobiont virulence and infection, but antibiotic-resistant bacteria are still a challenge associated with antimicrobial interventions [27]. Under some conditions, the adverse effects of broad-spectrum antibiotics will disrupt the host immune response against infection. In one study, mice exposed to antibiotics combined with dextran sodium sulfate (DSS) co-treatment developed a sepsis-like disease and not colitis. This process involved a decrease in beneficial bacterial species and primarily the expansion of pathobionts into the systemic sites [28]. However, a prior study demonstrated that antibiotic pre-treatment rendered mice more susceptible to DSS treatment, but the disease onset was not associated with the expansion of pathobionts into systemic sites; rather, it was due to a failure of gut epithelial injury repair via toll-like receptor (TLR)-dependent signals [29]. Normally, the recognition of TLR ligands on commensals by TLRs expressed on the intestinal epithelium results in a protective effect to maintain gut homeostasis [30]. Zeng *et al*. reported that gut symbionts can regulate systemic immunity. They demonstrated that the antigens highly expressed on the outer membrane of gram-negative bacteria induce systemic serum immunoglobulin (Ig) G production and that the microbiome-specific IgG plays a protective role in systemic infection [31]. Thus, gut microbiota and the derived products have systemic influences on extraintestinal tissues and organs. These findings suggest potential mechanisms by which gut commensals combat systemic infection.

Numerous studies have highlighted the pivotal roles of microbiota and their metabolic products in maintaining host immune and metabolic homeostasis [32,33]. Dietary fiber alters the composition of gut microbiota, such as *Akkermansia* and *Lachnospiraceae*, reducing systemic inflammation and improving survival [34]. Bacterial fermentation of dietary fiber can produce SCFAs, which have been observed to exhibit anti-inflammatory effects [21]. During *Klebsiella pneumoniae* infection, gut microbiome-derived SCFAs promote macrophage bacterial clearance by upregulating late endosomal/lysosomal adaptor, mitogen-activated protein kinase (MAPK) and MTOR activator 2 (LAMTOR2) [35]. Granisetron, which is derived from gut microbial metabolism, has anti-inflammatory effects, modulates the host immune system and mediates susceptibility to sepsis in mice [36]. Bacterial diversity influences immune regulation and disease outcomes; accordingly, one study found that mice obtained from different vendors had significantly different immunophenotypes and microbiome profiles; they even exhibited different responses in the context of sepsis. After cohousing, the mice had similar gut microbiome compositions and all immunophenotypical differences disappeared [37]. Interestingly, the gut microbiome also influences serum IgA, which depends on T-cell abundance, to protect against sepsis [38]. IgA-mediated crosslinking enchains bacteria in the gut to protect against pathogenic infection [39]. Research on the precise mechanism underlying the microbiome-mediated shaping of the immune system will be of good clinical translational value.

#### Gut barrier function in sepsis

The gut tract is colonized by trillions of microorganisms, and gut barrier integrity is crucial for preventing bacteria and bacterial products from translocating into the systemic circulation. Intestinal epithelial cells form a physical and chemical barrier that maintains intestinal homeostasis [40]. Claudins, occludin, junctional adhesion molecules and cytosolic proteins [e.g. zonula occludens (ZO)-1, ZO-2 and ZO-3] are essential tight junction proteins for barrier function [41]. Peyer’s patches, the lamina propria, mesenteric lymph nodes and intraepithelial lymphocytes constitute the largest ‘immune organ’ of the body. Some specialized epithelial cells that secrete mucins and antimicrobial proteins also regulate the immunological environment [40]. Critical illness could markedly induce gut barrier dysfunction and result in a leaky gut [42]. Severe burn injury is at high risk of developing sepsis and leading to multiple organ failure, and gut barrier dysfunction may play an essential role in this progression [43]. Moreover, burn injury has been reported to alter the gut microbial composition and decrease the concentration of SCFAs, which may disrupt gut barrier function [44]. Fecal microbiota transplantation has been demonstrated as an effective way to restore microbiome diversity and increase gut barrier integrity in a murine model of burn injury [45]. During the development of polymicrobial sepsis, the redistribution of colonic tight junction proteins, including claudins 1, 3, 4, 5 and 8, leads to gut barrier dysfunction, meanwhile protein expression of claudin-2 is markedly upregulated [46]. Tight junction alterations appear as early as 1 h after sepsis onset, then microbial pathogens translocate from the intestine into the bloodstream and then pass through the liver via the portal vein [47]. Sepsis results in intestinal hyperpermeability through the upregulation of inflammation, epithelial cell apoptosis and the alteration of microbiome composition [48,49]. Proinflammatory mediators, such as TNF-α, IL-1β, IL-6, are recognized as important factors for modulating the intestinal barrier function and increasing gut permeability [50–52]. Myosin light chain kinase (MLCK) is commonly associated with increased cytokine levels, which, in turn, activates MLCK and causes intestinal hyperpermeability [53]. Previous research has shown that gut hyperpermeability was prevented and the survival time was significantly increased in MLCK^−/−^ mice following cecum ligation and puncture (CLP) [54]. Intestinal epithelial cell (IEC) apoptosis is another phenomenon that occurs during sepsis [55]. When gut integrity is disrupted, the apoptotic response appears to be activated and IEC apoptosis may augment gut permeability [56]. The gut-specific overexpression of the anti-apoptotic protein, Bcl-2, has been shown to result in enhanced tight junction protein expression following sepsis-induced hyperpermeability [57]. Epidermal growth factor is a cytoprotective polypeptide, and mice administered with systemic epidermal growth factor, as well as those with enterocyte-specific overexpression of epidermal growth factor, display improved intestinal barrier function and survival following sepsis [58,59]. Alteration of gut microbial communities disrupts tight junctions and increases gut permeability [60], and reversal of gut barrier dysfunction can effectively decrease the mortality associated with sepsis [61].

Gut microenvironment changes have been reported to increase susceptibility to sepsis development. Translocating endotoxins and bacteria could activate systemic inflammation and promote organ dysfunction and failure [62]. Once barrier dysfunction has occurred, some other gut microbiome products, such as fecal-derived extracellular vesicles, are likely absorbed into remote organs via the lymphatic vessels and bloodstream [63]. Translocating bacteria can be phagocytosed by mesenteric lymph nodes in normal circumstances. But host deficiencies and immunosuppression are probably among the risk factors leading to bacterial translocation-associated complications. Human studies have recognized that bacterial translocation appears to be associated with significantly higher rates of postoperative sepsis among surgical patients [64]. A healthy intestine would preclude the translocation of PAMPs to the systemic circulation and inhibit immune reactions. Additionally, the gut–lymph hypothesis suggests that gut-derived factors could translocate through the mesenteric lymph to distal organs and induce systemic inflammation [65]. Viable bacteria have been detected in sterile tissue, such as mesenteric lymph nodes, of gnotobiotic mice intragastrically administered with the whole cecal microflora of specific-pathogen-free (SPF) mice [66]. Using a ligated mesenteric lymph duct murine model, Badami *et al*. confirmed that factors derived from the gut and carried by the mesenteric lymph duct might contribute to death associated with critical illness [67]. Moreover, mesenteric lymph collected from animals after trauma and hemorrhagic shock was sufficient to induce acute lung injury when injected into rats [68], which confirmed that the gut–lymph pathway is a driving factor for host immune system activation. The details of the molecular mechanisms that contribute to sepsis-induced barrier function are complicated. Directly or indirectly targeting tight junctions is effective for preventing bacterial translocating into the bloodstream and improving survival following sepsis.

#### Gut–liver axis in sepsis

Gastrointestinal and liver function is intricately connected via the portal vein, and the gut–liver axis appears to have critical responses to sepsis [69]. The liver plays key roles in preventing and ameliorating the effects of systemic infections through bacterial clearance and production of inflammatory mediators [70]. Additionally, intestinal bacteria can convert liver-derived bile acids and modulate host metabolism and immunity [71,72]. Hence, the interaction between hepatic function and microbial function may provide an important defense mechanism against pathogens via immune activation. Tissue-resident macrophages, Kupffer cells, are the first-line immune cells in the liver that defend against blood-borne bacteria. Quick blood clearance can prevent bacteremia and ensure a sterile bloodstream [73]. Kupffer cells can efficiently clear circulating microbiome-derived products depending on the macrophage complement receptor immunoglobulin (CRIg) [74]. Additionally, CRIg also recognizes and binds to the lipoteichoic acid of gram-positive bacteria to capture circulating pathogens [75]. The commensal metabolite, D-lactate, reaches the liver through the portal vein and promotes Kupffer cells to capture and kill pathogens from the circulation [76]. Liver-mediated bacterial capture is critical for limiting the dissemination of bacteria into the systemic circulation and other susceptible organs during infection. *Listeria monocytogenes* infections could induce the early necroptotic death of Kupffer cells, which induces hepatocytes to release IL-33, which promotes IL-4 production by basophils. Importantly, these, in turn, cause monocyte-derived macrophage recruitment to the liver with the activation of antibacterial inflammatory reactions, eventually restoring hepatic immune homeostasis [77]. As polarization of Kupffer cells plays an essential role in modulating their immune function in inflammation, enhancing the M2 (alternative) polarization phenotype could protect against liver injury [78]. Therefore, it is clear that sepsis results in liver injury, which further leads to host immune reaction disruption and increases susceptibility to infection. Future investigations will be required to explain the detailed turnover mechanism of liver macrophages upon infection leading to sepsis. More importantly, hepatocytes, hepatic stellate cells and sinusoidal endothelial cells may all be involved in immune activation during sepsis [79].

#### Bacterial outer membrane vesicles in sepsis

Bacterial outer membrane vesicles (OMVs) are naturally derived from bacteria, mainly from gram-negative bacteria [80]. OMVs contain many microorganism-associated molecular patterns (MAMPs), such as proteins, lipids, DNA and RNA, as well as abundant LPS. These MAMPs can recognize host pattern recognition receptors, leading to an immune response [81]. LPS, as a key outer membrane component of gram-negative bacteria, can be delivered via OMVs. OMVs isolated from *Escherichia coli* could trigger a sepsis response [82]. Moreover, OMVs activate the cytosolic LPS-sensing pathway during infection [83]. OMVs can act as a vehicle for delivering cargo into cells. They facilitate host–microbial crosstalk and enable bacteria to modulate intracellular communication within the host. Increased intestinal permeability also influences the delivery of OMVs from the gut into the circulatory system and remote organs, eventually causing systemic inflammation and sepsis [63]. Fecal-derived extracellular vesicles, which are secreted by multiple gut microbes, have the potential to induce peritonitis and sepsis-like systemic inflammation [63]. However, some OMVs also have beneficial effects on the host. Immunization with *Acinetobacter baumannii-*derived OMVs increased survival in a sepsis model [84]. Moreover, immunization with *Burkholderia pseudomallei-*derived OMVs can also provide protection against lethal sepsis, probably because OMVs may serve as a potent ‘vaccine’ [85]. Additionally, through the activation of Th1 and Th17 cells, *E. coli-*derived OMVs may prevent bacteria-induced mortality [86]. During infection, OMVs have many other effects on host cells, such as immune cells, endothelial cells and platelets [81]. However, the detailed mechanisms underlying the various effects of OMVs in sepsis have not been thoroughly studied. OMVs also need to be investigated for their therapeutic potential in the clinical treatment of sepsis.

#### Bacterial circadian rhythms in sepsis

Modern lifestyles, including jet lag and shift work, often cause circadian disruptions, which strongly influence host–microbial interactions with increased susceptibility to metabolic syndrome and gastrointestinal pathology [87]. Although there is no direct evidence demonstrating that the circadian rhythm of the microbiome could directly impact sepsis patients, several clues suggest that such an association might be possible. First, human gut microbiota display circadian oscillations, and such diurnal oscillations indeed directly influence host pathophysiology. For example, Thaiss *et al*. reported that dysbiosis induced by jet lag could disrupt glucose metabolism [88]. Second, in mice, *Salmonella enterica* infection with onset in the morning was shown to cause greater inflammation and pathogen colonization compared with night-onset infection [89]. This rhythmic difference may result from the crosstalk among the gut microbiome, host circadian system and immune responses [90]. Third, at the molecular level, gut microbiota-derived MAMPs could directly regulate host immune responses, especially in inflammatory disease development. All of these observations suggest that the gut microbiome may directly impact host immune responses during infection, and disruption of gut microbial circadian oscillation may, in turn, impact sepsis development.

Both the compositional and functional components of the gut microbiota exhibit robust circadian rhythms [91], and this has been recognized as a key modulator of host circadian rhythms [92]. Host sensitivity to a variety of infections follows circadian features such that inflammation levels are increased and susceptibility to pathogens is higher at the beginning of the resting phase [93]. Circadian disruption impacts the immune system and mediates the higher susceptibility to inflammatory disease [94]. It has been reported that dynamic changes in microbiome compositions have been observed in intensive care unit patients during the acute phase [95]. A light/dark cycle improved survival after CLP compared with a dark/dark cycle in a murine model [96]. Through the gut microbiota–subdiaphragmatic vagus nerve axis, short-term sleep after LPS administration has been shown to augment the systemic inflammatory reaction and organ injury associated with sepsis [97]. Importantly, host circadian disruption also affects the diurnal microbial fluctuations that accompany bacterial dysbiosis [88]. Rodents with disrupted circadian rhythms have been shown to exhibit dysbiosis with decreased levels of butyrate producers, such as *Eubacterium plexicaudatum* and *Subdoligranulum* [98]. Circadian rhythm disturbances decrease host radioresistance, and this may be partly mediated by gut microbiome composition [99]. Sleep deprivation could affect immune cell abundance and function, indicating that people with sleep loss may be more susceptible to infections [100]. These phenomena reveal the potential crosstalk between bacterial circadian rhythms and critical illness. Dysbiosis disrupts gut homeostasis, impacts the sensing pathway of commensal bacterial products and influences the circadian clock guiding epithelial cells and systemic metabolism [30]. Based on the circadian interactions of the host and microbiome, developing specific pharmacologic compounds aimed at regulating daily oscillations might be beneficial to host immune response modulation. Understanding the tight regulation between circadian rhythms, gut microbiota and the immune system will facilitate the development of more precise therapies to treat infection.

### Targeting the microbiome in sepsis therapy

Current clinical treatments for sepsis include the administration of antibiotics and nutritional support. However, antibiotics produce a sustained effect on the gut microbiome and may cause antibiotic resistance. Antibiotic resistance is still a challenge affecting the treatment of critically ill patients worldwide. Direct or indirect modulation of the gut microbiota may yield new potential therapeutic approaches to improve sepsis management.

**Table 1:** Some selected probiotics/prebiotics usage in clinic

Probiotics/prebiotics	Study population and intervention	Functions	References
Galacto-oligosaccharide mixture	• 65-80 years old volunteers • 10 weeks with daily doses of 5.5 g/d with a 4-week washout period in between	• Increase bacteroides and bifidobacteria • Affect immune function associated with ageing	[113]
*Lactobacillus plantarum* plus fructooligosaccharide	• Newborns • Beginning on day 2–4 of life for 7 days	• 40% reduction in the primary combined outcome of death and neonatal sepsis	[114]
*Bifidobacterium breve* strain Yakult and *Lactobacillus casei* strain Shirota plus galactooligosaccharides *Lactobacillus acidophilus*, *Enterococcus faecium* and *Bifidobacterium infantum* *Lactobacillus reuteri* DSM 17938 Multispecies probiotic^a^	• Ventilated patients with sepsis • Initiated within 3 days after admission • Preterm infants • twice daily until discharge • Preterm infants • once daily until discharge • Early sepsis patients • Twice daily for 4 weeks	• Increase the levels of beneficial bacteria and SCFAs • Decrease the incidence of enteritis and ventilator-associated pneumonia • Decrease the frequency of late-onset sepsis and infections • Improve feeding tolerance, promote growth • Increase daily defecations • Shorten the length of hospitalstay • Enrich the gut microbiome with probiotic strains • Increase gut functional diversity	[115] [116] [117] [118]

*SCFAs*: short-chain fatty acids

^a^Multispecies probiotic contains *Lactobacillus plantarum* W1, *Lactobacillus paracasei* W20, *Bifidobacterium bifidum* W23, *Lactobacillus salivarius* W24, *Lactobacillus acidophilus* W37, *Bifidobacterium lactis* W51, *Enterococcus faecium* W54, *Lactobacillus acidophilus* W55, *Lactobacillus plantarum* W62, *Lactobacillus rhamnosus*W71

#### Fecal microbiota transplantation

Fecal microbiota transplantation (FMT) is a therapeutic method for restoring a normal intestinal environment via the administration of healthy donor feces. Living microorganisms from healthy donors are transferred to the recipients to rescue the disordered microbial ecosystem, suppress the activation of inflammatory reactions and prevent pathogen colonization [101]. FMT has been applied in the treatment of many diseases, such as IBD, cancer and CDI [102–104]. In the treatment of antibiotic-resistant CDI, FMT has been demonstrated as more effective than vancomycin [105]. FMT has been successfully used to treat sepsis and improve clinical outcomes following microbiota composition and immune system modifications [106,107]. FMT restores the specific bacterial populations, drives the clearance of systemic pathogens and increases survival in sepsis. Importantly, the expression of interferon regulatory factor 3, which is necessary for TLR signaling pathway activation, has been shown to be altered by FMT [108]. Additionally, FMT induces the expression of gut tight junctions and improves the survival of rats with sepsis [61]. Using a murine model, researchers have shown that FMT modulates the intestinal microbiome composition; specifically, FMT has been shown to increase the abundance of commensals and reduce the abundance of opportunistic gut pathogens, alleviating septic encephalopathy via anti-inflammatory effects [109]. The clinical potential of FMT is promising. However, due to the complexity and variability of donor feces, the detailed mechanisms by which bacterial strains or metabolites are responsible for FMT’s beneficial effects are still not clear. Specialized methods, including capturing detailed donor medical data as well as standardized stool collection and preparation procedures, are required to reduce the risks associated with FMT. Collectively, the aim of FMT is to reconstitute a normal intestinal ecosystem to facilitate improved immune function against pathogens.

#### Probiotics, prebiotics and synbiotics

Restoration of microbial diversity is a complementary method for rebuilding the normal gut microbial ecology. Probiotic (‘good’ bacteria) supplementation has been shown to modulate the gut microbiome composition and yield beneficial effects on the host [110]. The beneficial roles of probiotics include immune modulation, pathogen prevention, gut barrier function improvements and some metabolic effects [110]. *Lactobacillus*, *Bifidobacterium* and *Streptococcus* are commonly used probiotics [111]. Prebiotics, as non-digestible nutrients, can promote commensal bacterial growth. Additionally, the administration of synbiotics, which are combinations of probiotics and prebiotics, regulates the host gut microbiome [112].

There are several published case reports detailing the clinical effects and outcomes of probiotics and prebiotics administration [113–118] (Table 1). In a cohort of elderly volunteers, administration of galacto-oligosaccharides as prebiotics for 10 weeks regulated fecal bacterial populations and natural killer cell activity [113]. Probiotics modulate gene expression in inflammatory pathways and the immune system in epithelial and immune cells, including nuclear factor of activated B cells protein kinase (NF-κB), MAPKs, IL-6, IL-8 and TNF-α. [119]. Administration of a synbiotic containing *Lactobacillus plantarum* has been associated with significantly decreased rates of neonatal sepsis and death [114]. A meta-analysis showed that supplementation of probiotics significantly reduces the risk of late-onset sepsis from 16.3% among placebo recipients to 13.9% among probiotic recipients [120]. Administration of probiotics effectively decreased mortality by regulating the microbiome composition and metabolites in a CLP mouse model [121,122]. A randomized controlled pilot trial revealed that probiotic intervention successfully increased stool bacterial diversity in early sepsis [118]. Singer *et al*. revealed that maternal antibiotic exposure affects neonatal susceptibility to late-onset sepsis, but some *Lactobacilli* strains can act as effective probiotics to prevent late-onset sepsis in susceptible individuals [123]. Furthermore, prophylactic *Lactobacillus rhamnosus GG* treatment improves gut barrier integrity and attenuates inflammatory responses in sepsis [124].

However, the efficacy and safety of probiotics are still controversial in clinical settings. In one study, translocation of probiotics from the mucosal epithelium to extraintestinal sites was rarely observed in healthy individuals, and even if it occurred, it hardly caused detrimental effects [125]. Such translocation may be limited by mesenteric lymph nodes or mediated by the macrophage killing function. However, probiotic administration is associated with an increased risk of bacteremia via probiotic bacteria transmission to the bloodstream among intensive care unit patients [126]. Cancer, diabetes and transplantation could increase the patients’ susceptibility to *Lactobacillus* bacteremia, mostly due to immunocompromise [127]. McNaught *et al*. conducted a prospective study of surgical patients and found that the mortality rate associated with sepsis was slightly higher in the probiotic group than the control group, although this difference was not statistically significant [128]. In another study, patients with non-severe sepsis undergoing probiotic treatment exhibited higher mortality risk [129], and another study showed that probiotics administration increased bacterial translocation in patients with organ failure [130]. These observations highlight the need to determine the specificity and safety of various strains used in sepsis treatment.

#### Phage therapy

Bacteriophages, also known as phages, are viruses within bacteria and archaea. Phages have anti-inflammatory and immunomodulatory effects [131,132]. Phages containing antibacterial agents are considered superior to antibiotics [133,134]. Bacteriophage therapeutics have been emerging as a novel approach for treating bacterial infections and their efficacy has been demonstrated in mouse models of sepsis [135,136]. In one study, a phage cocktail was tested against bacterial species isolated from neonates with sepsis [137]. In a single-arm, non-comparative trial, treatment with bacteriophages (AB-SA01) was safe for treating septic shock caused by *Staphylococcus aureus* infections [138]. Interestingly, lytic phages have been reported to knock down their targeted bacteria, affect other non-targeted bacterial communities and eventually modulate the gut metabolome [139]. Characterizing the interplay between phage and commensal bacteria will provide more precise approaches for infectious disease therapy. Understanding phage biology and acquiring the highly purified phages will pave the way for the development of phage therapeutics for sepsis. However, virus treatment still has potential risks and more reliable safety assessments are urgently needed.

## Conclusions

With the use of sequencing approaches, we can better comprehend the symbiotic crosstalk between microbiota and the host. Gut microbiota and associated products regulate key host functions. As highlighted in this review, sepsis induces gut microbial dysbiosis. Moreover, the gut microbiome participates in the development of sepsis and affects host susceptibility to sepsis. Additionally, gut–liver crosstalk may supply a basis for the treatment of sepsis-induced organ injury. Strengthening gut barrier function is a good method for minimizing translocation of bacteria and bacterial metabolites and alleviating sepsis-induced organ injury. The gut microbiome includes bacteria, fungi, viruses and archaea. The detailed roles of these microorganisms in the pathophysiology of sepsis need further investigation. Current limitations relate to whether gut microbiome composition can be used as a biomarker for sepsis outcomes. Hopefully, a more detailed understanding of the mechanistic role of gut microbiota in sepsis will allow for the development of original and effective approaches to alleviating sepsis morbidity and mortality.

## Abbreviations

CDI: *Clostridioides difficile* infection; CLP: cecum ligation and puncture; CRIg: complement receptor immunoglobulin; DAMPs: damage-associated molecular patterns; DSS: dextran sodium sulfate; FMT: fecal microbiota transplantation; HMGB1: high mobility group box 1 protein; IBD: inflammatory bowel disease; IECs: intestinal epithelial cells; Ig: immunoglobulin; IL: interleukin; LAMTOR2: late endosomal/lysosomal adaptor, MAPK and MTOR activator 2; MAPK: mitogen-activated protein kinase; MLCK: myosin light chain kinase; LPS: lipopolysaccharide; MAMPs: microorganism-associated molecular patterns; NF-κB: nuclear factor of activated B cells protein kinase; OMVs: outer membrane vesicles; PAMPs: pathogen-associated molecular patterns; PRRs: pattern-recognition receptors; SCFAs: short-chain fatty acids; SOFA: Sequential (or Sepsis-related) Organ Failure Assessment; SPF: specific-pathogen-free; TNF: tumor necrosis factor; TLRs: toll-like receptors; ZO: zonula occludens.

## Authors’ contributions

M.N. drafted the manuscript, P.C. supervised and edited the review.

## Funding

This study was supported by National Natural Science Foundation of China (81 873 926) toPC.

## Conflict of interest

The authors declare no conflict of interest.
